# The Canine Hookworm *Ancylostoma Caninum*: First Confirmed Evidence in a Dog in Central Europe: Epidemiological Relevance or Coincidence?

**DOI:** 10.3390/pathogens14121241

**Published:** 2025-12-04

**Authors:** Michaela Liptáková, Andrea Schreiberová, Zuzana Cellengová, Viktória Kožárová, Gabriela Štrkolcová

**Affiliations:** 1 Department of Epizootiology, Parasitology and Protection of One Health, University of Veterinary Medicine and Pharmacy in Košice, Komenského 73, 040 01 Košice, Slovakia; michaela.kadukova@student.uvlf.sk (M.L.); andrea.schreiberova@uvlf.sk (A.S.); zuzana.cellengova@student.uvlf.sk (Z.C.); 2 Institute of Geotechnics SAS Košice, Watsonova 45, 040 01 Košice, Slovakia; viki.kozarova22@gmail.com; 3 Faculty of Materials, Metallurgy and Recycling, Technical University of Košice, Letná 9, 042 00 Košice, Slovakia

**Keywords:** *Ancylostoma caninum*, dog, molecular identification, zoonoses, larva migrans cutanea, climate change

## Abstract

Canine hookworms represent some of the most globally prevalent parasitic nematodes affecting dogs and pose a significant zoonotic risk to humans, in whom they can induce cutaneous larva migrans. Infection with these parasites may lead to blood loss, anaemia, and, in severe cases, mortality—particularly in young puppies. The present study reports a confirmed case of *Ancylostoma caninum* infection in a 15-month-old dog in the Slovak Republic. The infected dog presented with severe, persistent diarrhoea, and haematological examination revealed a decrease in erythrocyte and haemoglobin levels, as well as mild eosinophilia. Coprological examination confirmed the presence of nematodes belonging to the family of Ancylostomatidae. Following the initiation of anthelmintic therapy, adult individuals were recovered from the faeces. Based on distinct morphological characteristics, the parasites were presumptively attributed to the species *A. caninum*. Subsequent molecular analysis of the mitochondrial cytochrome c oxidase subunit 1 gene (COX1) and the ribosomal ITS regions definitively confirmed the species *A. caninum*. Our findings confirm that this is the first molecularly confirmed case of this species in Central Europe. This hookworm is predominantly found in warm and humid climatic regions. Its recent detection in Slovakia, a country characterised by a temperate climate, may suggest a northward expansion of its geographic range, potentially facilitated by ongoing climatic shifts associated with global climate change.

## 1. Introduction

Hookworms are the most common soil-transmitted helminths, which are widespread globally in populations of dogs, cats, and wild carnivores, posing a threat to public health [1,2]. Parasitic nematodes of the Ancylostomatidae family include the *Ancylostoma* and *Uncinaria* genera, which are important from the veterinary point of view as they are one of the key agents causing alimentary enteritis in carnivores. Carnivore infections are caused by five species: *Ancylostoma caninum*, *Ancylostoma ceylanicum*, *Ancylostoma braziliense*, *Ancylostoma tubaeforme*, and *Uncinaria stenocephala.* All of those species, except *A. tubaeforme*, exhibit zoonotic potential, while *U. stenocephala* is regarded as the species with a lower but still potential zoonotic risk of being transmitted to humans [3,4,5]. *A. caninum* is the most pathogenic species of hookworms infecting carnivores, primarily dogs. In cats, this infection occurs only exceptionally, mostly in isolated cases [6,7]. The parasite causes significant blood loss, leading to anaemia, haemorrhagic enteritis, and in the case of high infection intensity, especially in puppies, it may even cause death [3,8,9]. It is the species with cosmopolitan distribution, primarily in the tropical and subtropical countries of Africa, Asia, Australia, North and South Americas, as well as Europe. Its occurrence is limited especially to regions with warm climates and optimal humidity—conditions that are ideal for their development [2,8]. *A. ceylanicum* and *A. braziliense* parasitise animals of the Canidae and Felidae families, in tropical and subtropical regions of Asia, Africa, and South America [3,6,10]. *U. stenocephala* is regarded as a less pathogenic species. As to its geographical distribution, its occurrence has most frequently been reported from countries with a temperate climate, in particular from the northern regions of the United States, Canada, Europe, Africa, as well as Australia—New South Wales and Queensland regions—and New Zealand [3,4,11,12,13]. *A. tubaeforme* is an exceptional hookworm species with global occurrence, which strictly prefers the Felidae family, especially domestic cats, as hosts. Unlike the other species of the *Ancylostoma* genus, it does not pose a zoonotic risk and it has never been observed in dogs so far [3,14,15].

With regard to their life cycle, several transmission routes are known, the most frequent ones being the percutaneous and oral transmission routes. *A. caninum*, *A. tubaeforme*, and *A. braziliense* are primarily transmitted by infective L3 larvae penetrating the skin and entering the bloodstream, through which they are transported to the lungs through the heart. In the lungs, they penetrate into pulmonary alveoli and then migrate up the bronchial tree to the pharynx, where mucosal irritation causes the larvae to be coughed up and subsequently swallowed by the host. They continue their development in the host’s digestive tract. In the intestine, larvae undergo moulting and develop into L4 and L5 larvae and then adults; using their buccal capsule, they attach to the intestinal mucosa and feed on the host’s blood. Adult females produce eggs, which are passed in the host’s faeces into the external environment [4,10,16,17]. The eggs of the *A. caninum* species are 58–76 µm long and 36–41 µm wide, while the eggs of *A. braziliense* are sized 58–76 µm × 34–39 µm, and the eggs of *A. tubaeforme* are sized 58–66 µm × 38–42 µm [3]. Compared to the aforesaid eggs, the eggs of *U. stenocephala* are larger—with a length of 71–92 µm and a width of 35–58 µm [2,3]. In damp and warm soil, eggs develop into first-stage (L1) larvae in 1–2 days. The larvae then undergo moulting twice, and in 5–10 days, they develop into the infective filariform L3 stage [3,4,10,16]. Alternatively, their oral transmission is also possible, but less frequent. Infection typically begins after a host intakes contaminated soil, grass, or water [16]. Following the oral intake of filariform larvae and their passage through the digestive tract, larvae may either mature directly in the intestine or penetrate through the oral mucosa and then migrate in a manner similar to percutaneous transmission. Subsequently, adult individuals develop after a short time spent in the intestinal wall [9,10,16]. *A. caninum* is the only species known to be capable of transmammary transmission to puppies [3,18]. Infection is a result of the reactivation of hypobiotic larvae in the tissues of pregnant bitches. Subsequently, the larvae pass into the mammary glands, from where they are transmitted in colostrum and milk to newly born puppies for approximately 18 days, while the most intensive excretion of larvae has been observed during weeks 1 and 2 [19,20,21]. L3 larvae, ingested by puppies together with breast milk, are then released in the digestive tract, enter the bloodstream, and migrate to the lungs, where they penetrate the alveoli. Their further development is identical to the course of percutaneous infection—larvae migrate up the bronchial tree to the pharynx, and they are coughed up and subsequently swallowed. In the small intestine, they mature into adult individuals [16,19,20,21]. The highest blood loss in dogs per worm in the Ancylostomatidae family was observed for the *A. caninum* species, in particular, as much as 10–200 µL. *A. ceylanicum*, *A. braziliense*, and *U. stenocephala* caused significantly lower losses, amounting to 14 µL, 1 µL, and 0,3 µL, respectively [22]. While older publications described a possibility of transplacental transmission of *A. caninum*, newer studies, conducted over the last decade, refuted that hypothesis [2,4,23,24]. Transmission is also possible by eating a paratenic host, mainly a rodent [2,18,25].

Canine hookworms cause *Cutaneous Larva Migrans* (CLM) syndrome in humans. Symptoms induced by CLM begin with the presence of erythematous and itchy papules at the site where the larvae penetrated the skin [10,26,27]. Larvae typically migrate between the epidermal layers, most frequently between the stratum germinativum and stratum corneum [27,28]. In the literature, cutaneous manifestations were assigned various terms, and they are typically referred to as ‘creeping eruption’ or as linear, serpiginous (snakelike), erythematous, tortuous tracks associated with intense pruritus [3,10,29]. Among canine hookworm species, only the larvae of *A. ceylanicum* after percutaneous skin penetration in humans are capable of completing their full developmental cycle in humans; this means that adult worms develop in the small intestine and females are capable of producing eggs. This cycle typically runs without CLM [10,30]. There is evidence that *A. caninum* larvae are also capable of completing their life cycle in humans and causing eosinophilic enteritis, unlike larvae of *U. stenocephala* and *A. braziliense*, which do not reach adulthood [10,31].

In Central Europe, *U. stenocephala* is the most frequent hookworm species, regarded as the hookworm that is most widespread in carnivores in regions with a temperate climate [4,10,32,33,34,35]. As for *U. stenocephala*, the temperature range that is optimal for larval development is from 7 °C to 30 °C, while the optimal temperature is 20 °C; *A. caninum* larvae need temperatures ranging from 15 °C to 37 °C, with the optimal temperature being 30 °C [36,37]. In the years 1991–2021, the average annual air temperature in Europe increased by approximately 0.5 °C per decade [38]. Global warming has caused significant changes in the geographical distribution of multiple parasitic diseases. Rising temperatures and changes in the precipitation quantities and frequency contribute to the formation of conditions that support the spread of vectors like mosquitos and ticks; as a result, parasites like *Dirofilaria* spp. and *Babesia* started to be present in regions where their presence had not been previously observed. A similar trend may also be detected for soil-transmitted helminths, since the climate factors, such as temperature and humidity, significantly affect the developmental cycles of parasites and survival of their infective stages in the environment, and hence also their endemic occurrence [39,40,41,42,43]. The occurrence and spread of hookworms in Europe are also linked to rising temperatures and humidity. Climate change alters the distribution and life cycles of parasites and their hosts, supporting the possibility of hookworm expansion into previously temperate regions [44,45].

Previous studies, conducted in Slovakia, indicate that the eggs of the Ancylostomatidae family frequently infect domestic and shelter dogs [5,46,47,48,49]. Molecular analysis carried out in Slovakia in 2022 confirmed the presence of the *U. stenocephala* species in dogs [5]. This study is a clearly confirmed case of infection with *Ancylostoma caninum* in a 1.5-year-old female dog in Slovakia, representing the first such case reported in Central Europe. The question arises as to whether the distribution of *Ancylostoma* spp. in Central Europe is changing as a result of climate change and whether these findings indicate an eastward expansion of hookworms. Moreover, the role of wild carnivores, such as foxes, as well as domestic cats, as potential reservoirs of *Ancylostoma* spp. in natural environments remains insufficiently explored. However, information on the current distribution and molecular identification of hookworm species in Europe remains limited.

## 2. Materials and Methods

### 2.1. Case Presentation

In this article, a clinical case of a 15-month-old bitch of the Neapolitan Mastiff breed is presented. The animal was examined at a private veterinary clinic in Košice for epileptiform seizures experienced over the previous three months. The patient’s weight was 50 kg, and it came from a Hungarian pig farm at a location unknown to the owner. At the time of examination, the animal had been living with the new owner for three months, in an apartment in the city of Košice (48°43′15″ S 21°15′28″ V). The medical history also indicated that the animal had been vaccinated against rabies (Nobivac Rabies, Intervet International B.V., Boxmeer, The Netherlands) and against infectious canine diseases (Nobivac DHPPi and Nobivac L4, Intervet International B.V., Boxmeer, The Netherlands). It was also dewormed once using a combined preparation against internal and external parasites, containing afoxolaner and milbemycin oxime as active substances (Nexgard Spectra, MERIAL, Toulouse, France). Clinical examination, conducted at a private clinic, did not reveal any significant abnormalities, and no pathological findings were observed in biochemical blood tests, while haematological blood tests indicated a slight decrease in the erythrocyte count and haemoglobin level, as well as mild eosinophilia with a value of 1.70 × 10^9^/L. However, the rest of the haematological parameters were free of any pathological findings. The patient underwent not only standard examination but also blood sample collection for the purpose of identifying a blood ammonia level, while its value was identified as x = 24 μmol/L (N < 50.0 μmol/L), and serum collection for the purpose of identifying antibodies against *Toxoplasma gondii.* Using the blood serum sample, IgG antibodies against *T. gondii* were identified in a quantity of 250 IU/mL (values above 55 IU/mL are regarded as a positive result). Subsequently, the patient underwent 4-week antibiotic therapy with clindamycin as an active substance acting against *T. gondii*, during which no additional seizures were observed. However, after the therapy was completed, the patient experienced persistent diarrhoea episodes, accompanied by a distinctive smell. In order to exclude a parasitic cause, its faeces were subjected to coproscopical examination at the Department of Epizootiology, Parasitology and Protection of One Health.

### 2.2. Parasitological Examination

#### 2.2.1. Examination of Samples by Applying the Faecal Flotation Method

Samples were analysed for the presence of oocysts, cysts of protozoa, and eggs of helminths by applying the faecal flotation method, which included the use of a flotation solution containing magnesium sulphate and sodium chloride with a specific gravity of 1.24 g/cm^3^. Zinc sulphate solution with a specific gravity of 1.18 g/cm^3^ was used to detect cysts of *Giardia duodenalis.* Subsequently, the samples were analysed under a light microscope [50].

#### 2.2.2. Coproculture Technique and Morphological Identification

**Figure 1:**
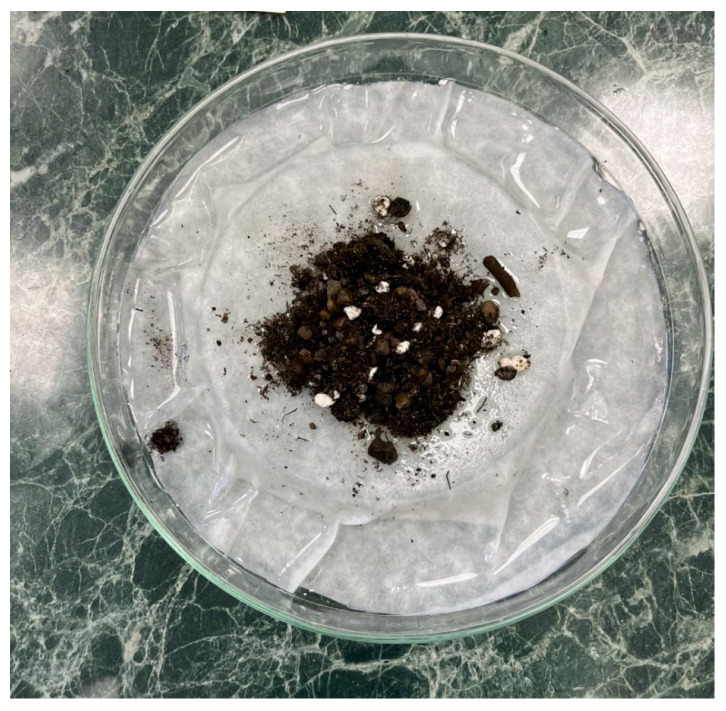
Coproculture method.

Samples with a positive finding of the strongyle eggs of the Ancylostomatidae family were further subjected to a coproculture analysis by applying the coproculture technique modified by Garcia and Bruckner [50]. The technique consisted of placing a small Petri dish into a larger one, thus creating an island, which was then covered with a large sheet of filtration paper. After water was added, it was ensured that the edges of the filtration paper were immersed. Approximately 3 g of faeces, 5 g of sterile soil, and 1 mL of sterile water were thoroughly mixed and applied onto the filtration paper covering the island. The Petri dish was then closed and sealed with parafilm (Parafilm M laboratory film, BEMIS). After 7 days of incubation at a temperature of 25–28 °C, the eggs developed into larvae of various stages. They were morphologically identified using the key presented in a paper by Gibbs et al., (1961) (Figure 1) [51]. Following the therapy initiation, it was possible to extract adult individuals from the faeces. They were subsequently subjected to morphological identification. All microscopic images and measurements of larval stages and adult individuals were made using light microscopy and PROMICRA QuickPHOTO 3.0 Microscopy Imaging Software. The images redrawn from the original photographs were created using Adobe Photoshop software version 27.1.

#### 2.2.3. Molecular Identification and Phylogenetic Analysis

Genomic DNA was extracted from two samples with larvae and six adult nematodes collected from this study, using a commercial DNeasy^®^ Blood and Tissue kit (Qiagen, Hilden, Germany) according to the manufacturer’s protocol. Molecular identification of nematodes was performed by PCR amplification and sequencing of two different DNA regions—cytochrome c oxidase subunit 1 gene (COX1) of mitochondrial DNA and based on an analysis of ITS regions of ribosomal DNA (includes partial sequence ITS 1; 5.8S ribosomal RNA gene, complete sequence; and ITS 2, partial sequence). A partial fragment of the mitochondrial gene COX1, approximately 710 base pairs in length, was amplified by PCR using universal primers for metazoan invertebrates: forward primer LCO1490 (5′-GGTCAACAAATCATAAAGATATTGG-3′) and reverse primer HCO2198 (5′-TAAACTTCAGGGTGACCAAAAAATCA-3′) [52]. The PCR thermal cycling conditions were as follows: initial denaturation at 94 °C for 3 min; followed by 5 cycles of 94 °C for 30 s, 45 °C for 30 s, and 72 °C for 1 min; followed by 35 cycles of 94 °C for 30 s, 51 °C for 1 min, and 72 °C for 1 min; and a final extension at 72 °C for 10 min [53]. The more variable ITS regions of ribosomal RNA genes were amplified using universal primers for nematodes (forward primer NC16: 5′-AGTTCAATCGCAATGGCTT-3′ and reverse primer NC2: 5′-TTAGTTTCTTTTCCTCCGCT-3′), producing an amplicon of approximately 1250 bp. The PCR conditions were as follows: 94 °C for 5 min; 30 cycles of 94 °C for 30 s, 55 °C for 30 s, and 72 °C for 1 min; followed by a final extension at 72 °C for 5 min [54]. The PCR products of all samples (cox1 and ITS regions) were loaded onto 1% agarose gels prepared in TAE buffer and stained with GoodView^TM^. Electrophoresis was carried out for 40 min at 100 V, and DNA was visualised under a UV transilluminator. All positive PCR products were purified and sequenced in both directions by the commercial laboratory SEQme (Dobříš, Czech Republic) using the same primers as for PCR. Sequencing was carried out using the Sanger method. The obtained sequences were edited in MEGA X and assembled with Gene Tool Lite 1.0 (BioTools Inc., Jupiter, FL, USA) [55]. Relevant sequences were compared with those available in GenBank using the BLASTn algorithm (https://blast.ncbi.nlm.nih.gov/Blast.cgi, accessed on 15 September 2025) The COX1 and ITS sequences generated in this study were deposited in GenBank under unique accession numbers PX312830-PX312837, URL (accessed on 15 September 2025) and PX446876-PX446880 URL (accessed on 11 October 2025), respectively. For phylogenetic analysis of the mitochondrial COX1 gene, sequences of *Ancylostoma caninum* obtained in this study (Slovakia) were analysed together with *A. caninum*, *A. ceylanicum*, *A. tubaeforme*, *A. duodenale*, and *Uncinaria stenocephala* sequences available in GenBank (NCBI) from other regions of the world. Sequence alignment was conducted, and a phylogenetic tree was reconstructed using the maximum likelihood method with the Tamura–Nei model. The phylogenetic tree with the highest log-likelihood (−2118.50) is presented. Initial trees for the heuristic search were automatically generated using the Neighbour-Joining and BioNJ algorithms based on pairwise distances estimated with the Maximum Composite Likelihood (MCL) method, and the topology with the highest log-likelihood value was selected. Bootstrap analyses were conducted using 1000 replicates. The analysis included 20 nucleotide sequences comprising 670 positions in the final dataset. Evolutionary analyses were conducted in MEGA X [55].

## 3. Results

### 3.1. Coprological Diagnosis

**Figure 2:**
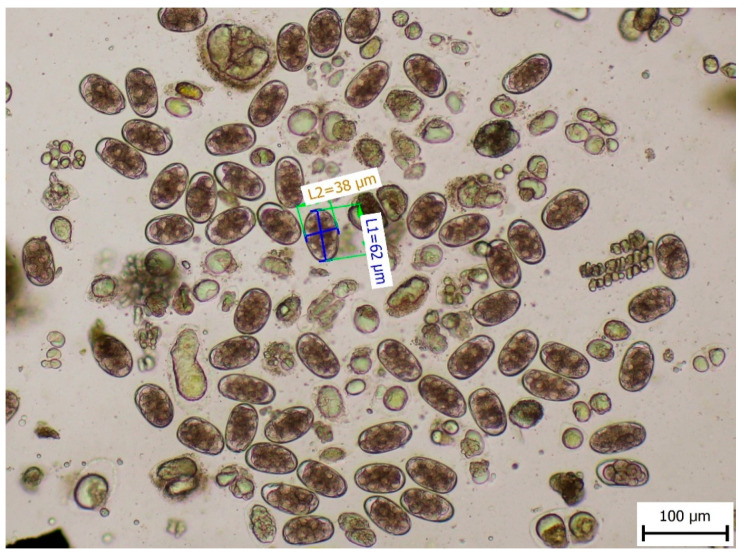
Length and width of the eggs of the Ancylostomatidae family.

Microscopic diagnosis of faeces confirmed the presence of eggs of the Ancylostomatidae family. Morphometric measurements of the eggs were made to identify their sizes (length by width) of 57–62 µm × 36–38 µm, which corresponded to the eggs of *A. caninum* (Figure 2).

### 3.2. Morphological Identification of L3 Larval Stage and Adult Individuals

**Figure 3:**
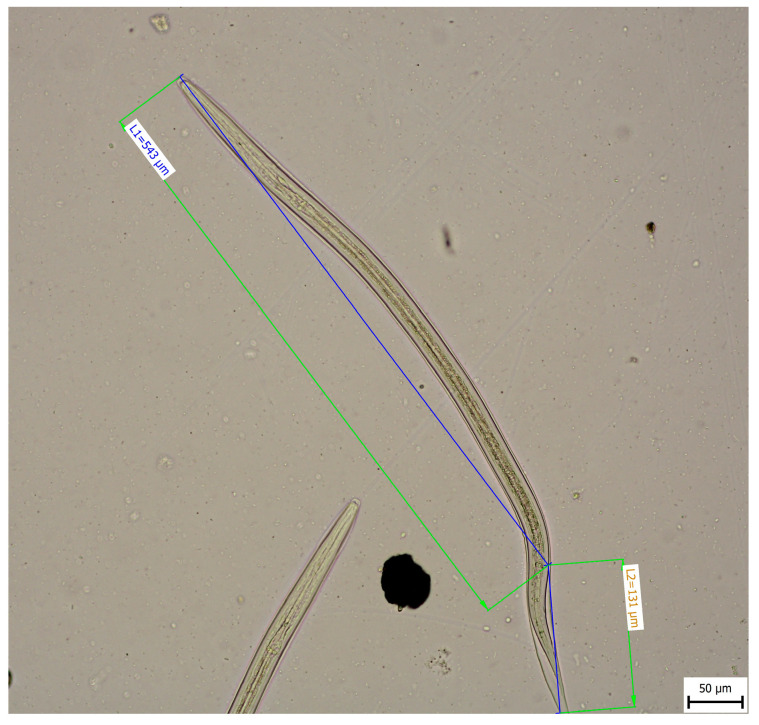
Larval stage L3.

**Figure 4:**
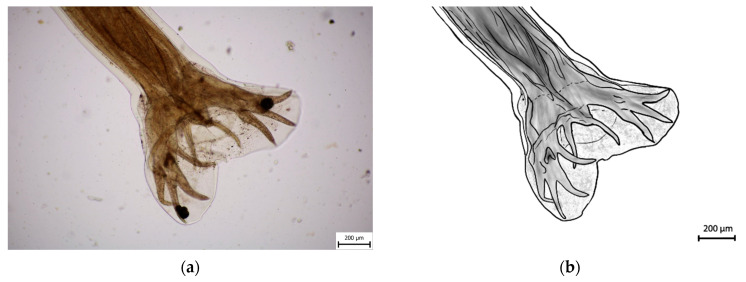
Adult male of *A. caninum*–posterior part: (**a**) Image obtained using light microscopy and PROMICRA QuickPHOTO; (**b**) redrawn image created using Adobe Photoshop software version 27.1.

**Figure 5:**
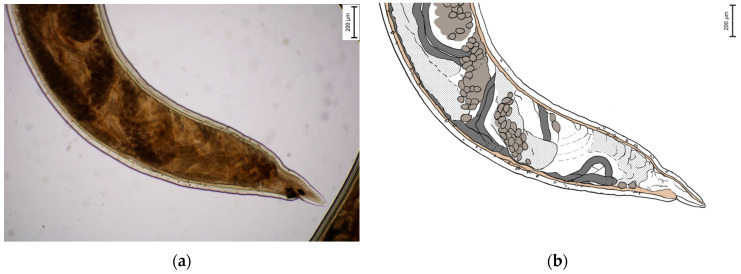
Adult female of *A. caninum*—posterior part: (**a**) Image obtained using light microscopy and PROMICRA QuickPHOTO; (**b**) redrawn image created using Adobe Photoshop software.

**Figure 6:**
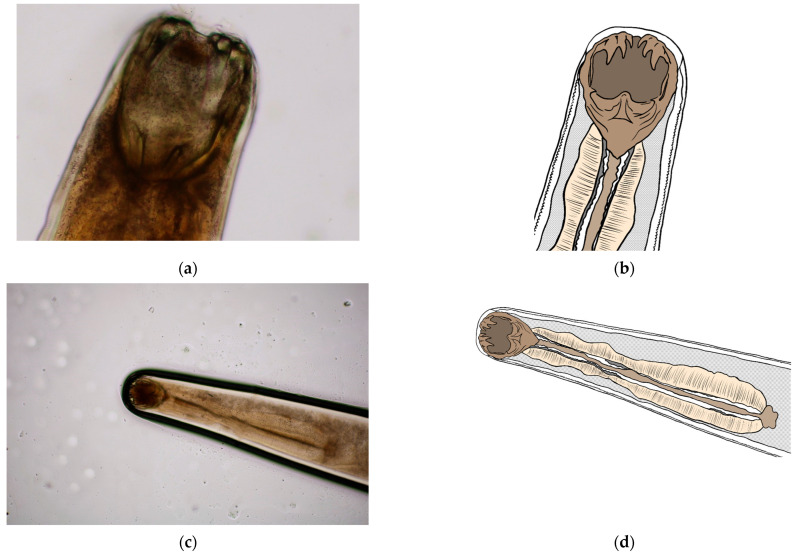
Buccal capsule of *A. caninum*: (**a**,**c**) Image obtained using light microscopy and PROMICRA QuickPHOTO; (**b**,**d**) redrawn image created using Adobe Photoshop software.

Morphometric analysis was conducted with L3 larvae obtained from the coproculture, while their lengths ranged from 590 to 674 µm (Figure 3), as well as adult larvae extracted from the faecal samples following the initiation of therapy. Adult individuals of both genders, males and females, were identified. Males were identified based on a well-developed bursa that ended with spine-like spicules positioned on three muscular rays (Figure 4). The males were smaller than the females. The females were identified based on the presence of the uterus filled with eggs, while the vulva was located in the junction between the middle third and the posterior third of the worm (Figure 5). The buccal capsules of adult individuals comprised three pairs of ventrally positioned teeth, which is the main morphological feature that distinguishes them from the *U. stenocephala* species. The image also shows the rhabditiform oesophagus (Figure 6). Based on the presence of specific morphological traits in adult individuals, they were unequivocally identified as *Ancylostoma caninum*.

### 3.3. Molecular and Phylogenetic Analysis

**Figure 7:**
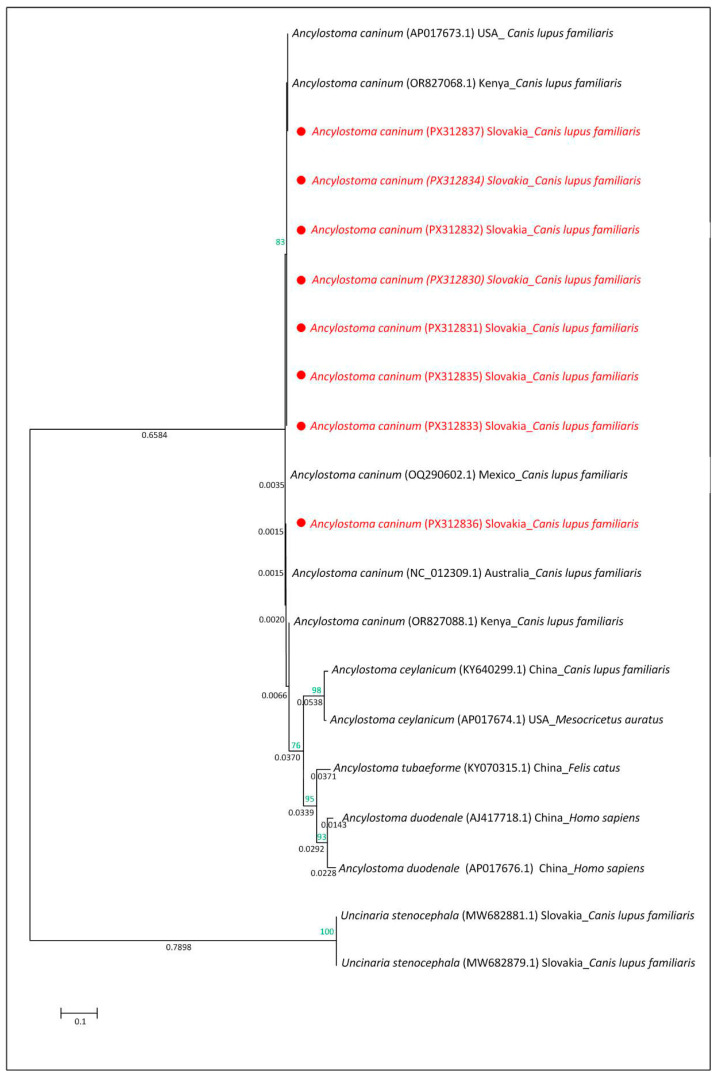
Evolutionary relationships based on mitochondrial COX1 gene sequences of species within the family Ancylostomatidae. The tree was reconstructed using the maximum likelihood method under the Tamura–Nei model. Bootstrap values (1000 replicates) are shown at the nodes (<50%). The tree is drawn to scale, with branch lengths proportional to the number of nucleotide substitutions per site (scale bar). Sequences obtained in this study (Slovakia) are marked with red.

Molecular analysis of the ITS regions and mitochondrial COX1 gene confirmed the presence of the zoonotic species *Ancylostoma caninum* in all positive samples. For the ITS regions of rDNA, five high-quality sequences (PX446876–PX446880) were obtained and compared using the BLAST tool in GenBank; they were a 100% match to the *A. caninum* KP844730.1 sequence (host: *Canis lupus familiaris* from Australia) and the OQ256235.1 sequence (host: *Canis lupus familiaris* from Mexico). Molecular analysis of the mitochondrial COX1 gene revealed that eight sequences obtained in this study (GenBank accession numbers PX312830–PX312837) showed 99.27–100% nucleotide identity with the reference sequence AP017673.1 of *A. caninum* (host: *Canis lupus familiaris*, USA: Baltimore). For the phylogenetic analysis, eight *A. caninum* mitochondrial COX1 sequences from this study (Slovakia; PX312830–PX312837) were analysed together with reference sequences from NCBI GenBank for *A. caninum*, *A. ceylanicum*, *A. tubaeforme*, *A. duodenale*, and *Uncinaria stenocephala* from various geographic regions and hosts. The following COX1 gene sequences from species of the family Ancylostomatidae were included in the phylogenetic analysis: *A. Caninum* AP017673.1 (host: Canis lupus familiaris from USA: Baltimore), OR827068.1 and OR827088.1 (host: Canis lupus familiaris from Kenya), OQ290602.1 (host: Canis lupus familiaris from Mexico), NC_012309.1 (host: *Canis lupus familiaris* from Australia; [56]); *A. ceylanicum* KY640299.1 (host: Canis lupus familiaris from China) and AP017674.1 (host: *Mesocricetus auratus* from USA; [57]); *A. tubaeforme* KY070315.1 (host: *Felis catus* from China; [14]); *A. duodenale* AP017676.1 (host: Homo sapiens from China) and AJ417718.1 (host: Homo sapiens from China; [58]) and U. stenocephala MW682881.1 and MW682879.1 (host: Canis lupus familiaris from Slovakia; [5]). Phylogenetic analysis of the COX1 gene revealed that *Ancylostoma caninum* clustered with *A. ceylanicum*, *A. tubaeforme*, and *A. duodenale*, whereas *Uncinaria stenocephala* formed a distinct branch on the phylogenetic tree. The *A. caninum* sequences from Slovakia clustered together and grouped *A. caninum* sequences from other geographic regions (Figure 7).

### 3.4. Treatment of A.caninum

Based on the results of coprological examination and therapy recommendations, the therapy initiated comprised a combination of the following active substances: febantel, pyrantel, and praziquantel (Dehinel Plus XL, KRKA d.d, Novo Mesto, Slovenia) at an oral dose of 15 mg of febantel, 4.4 mg of pyrantel, and 5 mg of praziquantel per 1 kg of live weight once daily for three consecutive days. In addition to the antiparasitic therapy, the patient was also administered probiotics (PURINA PRO PLAN FortiFlora Canine Probiotic, Nestlé Slovensko s.r.o, Prievidza Slovakia) throughout the entire therapy period with the aim of supporting its intestinal microflora. Also, the patient was on a gastrointestinal diet in the form of granulated feed (SPECIFIC CID Digestive Support). The result of the therapy was that the digestive symptoms were resolved; in particular, watery and smelly diarrhoea was mitigated. However, repeated coprological examination, conducted 10 days after the first dose of the antiparasitic drug, confirmed persisting infection. Therefore, the improvement in clinical symptoms was attributed to the adjuvant therapy. Subsequently, the selected therapy was a combination of fenbendazole, pyrantel, and praziquantel as active substances (Cestal Plus, CEVA ANIMAL HEALTH SLOVAKIA, s.r.o, Bratislava, Slovakia), administered at an oral dose of 20 mg of fenbendazole, 14.4 mg of pyrantel, and 5 mg of praziquantel per kg of live weight once daily for three consecutive days. After the therapy was completed, a check-up coprological examination was carried out (10 days after the administration of dose 1) with a negative result; moreover, digestive troubles completely subsided. However, according to medical records of later dates, 6 months after the therapy, the patient presented with recurrent clinical symptoms in the form of significantly smelly diarrhoea. Coproscopical examination of faeces for the detection of the eggs and molecular identification of third-stage (L3) larvae obtained from coproculture confirmed reinfection with *A. caninum.* Therapy combining fenbendazole, pyrantel, and praziquantel as active substances was initiated again, at an oral dose of 20 mg of fenbendazole, 14.4 mg of pyrantel, and 5 mg of praziquantel per 1 kg of live weight once daily for three consecutive days. The repeated administration of the same therapy had a positive effect and led to the complete recovery of the patient. Check-up coprological examinations, conducted 10 days, 2 months, and 5 months after the last therapy, confirmed the result.

## 4. Discussion

In the case described in this paper, a 15-month-old female dog was diagnosed with canine ancylostomosis based on its medical history, the clinical symptoms observed, as well as laboratory tests conducted (haematology, biochemistry, coprology, and morphological and molecular diagnosis). Based on the parasitological confirmation of eggs, in conjunction with haematological parameters, the bitch was confirmed to be presenting with anaemia and eosinophilia, indicating potential infection caused by the *Ancylostoma* genus.

In Slovakia, multiple studies, based on microscopic examination of faeces, were carried out; they investigated the general prevalence of parasites in dogs. A 2007 study indicated an 18.4% prevalence of eggs of *Ancylostoma/Uncinaria* spp. [59]. Later studies, conducted in 2017–2022 with dogs that lived in various living conditions, including dogs from shelters, dogs from marginalised settlements, and companion dogs from typical households, confirmed the presence of eggs of *Ancylostoma/Uncinaria* spp. amounting to 50.39%, 20.94%, 8.3%, 4.3%, 14.5%, and 6.3%, respectively [5,47,48,49,60,61]. Although *Ancylostoma/Uncinaria* spp. eggs were previously detected in dogs in Slovakia, the occurrence of *A. caninum* was unlikely under the cooler climatic conditions that historically prevailed in the region. However, recent increases in average temperatures, together with milder winters and longer warm periods, may have now created conditions favourable for its development and transmission.

Authors of several European studies have identified the Ancylostomatidae family as the most frequently occurring parasites in dogs living in diverse habitats [34,35,62,63,64,65,66,67]. Higher prevalence values were observed in Portugal—40.9%, Serbia—41.0%, and in another study from Serbia—40.1% [32,34,65]. In Spain, a study by Remesar et al., 2022, identified a 31.2% prevalence, while the prevalence values in Italy and Greece were significantly lower—9.16% and 9.4% [12,66,68]. A recent Polish study by Tylkowska et al., 2024, confirmed a prevalence of 11.5% [35]. Ukraine has a limited number of studies examining the prevalence of *Ancylostoma*/*Uncinaria* in dogs. A study by Ponomarenko et al., 2016, reports a prevalence of 44.4%, whereas Mushinskyi et al., 2024, report only 4.8% [69,70].

Despite only a limited number of studies, a number of papers dealing with the occurrence of hookworms in wild carnivores have been published. A study by Čabanová et al., 2017, conducted in Slovakia, confirmed the presence of eggs of the Ancylostomatidae family in wolves, ranging from 6.8% to 13.9% [71]. Other European studies have reported that hookworms were also detected in foxes, with a prevalence of 10–12%, and in wolves, with a prevalence of 30–90% [72,73,74]. This is a key factor, as wolves and foxes can migrate unrestricted across country borders, with foxes especially moving closer to human settlements, potentially leading to the introduction of various hookworm species into new regions where those parasites were previously not present [75]. Another factor may be the increasing occurrence of the golden jackal in Central Europe, which could serve as a potential reservoir of infection for red foxes and, consequently, for domestic dogs. Although the species occurs only sporadically in Slovakia, its dispersal has been documented across most neighbouring countries [76,77,78,79].

Even though the previous studies indicated a high prevalence of hookworms in Europe, references to molecular identification of those parasites in the literature are still limited. Štrkolcová et al. (2022), presented the first ever morphometrical and molecular confirmation of the U. *stenocephala* species in a dog in Central Europe. Genotyping based on the genes mt COX1, 18S rRNA, and ITS1 region confirmed the presence of the species in all positive samples [5]. In Europe, only two molecular studies have been conducted—in Italy—confirming the presence of the *A. caninum* species in dogs [80]. According to the available journals written in English, and based on the sequences listed in the GenBank, no other molecular studies confirming that species in Europe have been published.

In our study, two independent DNA regions were used to identify larvae and adult worms isolated from the dog faeces in eastern Slovakia. The mitochondrial COX1 gene, encoding subunit I of cytochrome oxidase, has long been recognised as a suitable molecular marker for evolutionary and barcoding studies across a wide range of organisms, including invertebrates, due to its high sequence variability and conserved primer sites [52,53]. Similarly, the more variable ITS regions of ribosomal RNA genes, amplified with universal nematode primers NC16 and NC2, provide high discriminatory power for species identification and phylogenetic analyses because they evolve faster than conserved rRNA genes such as 18S or 28S [54,76]. The use of both COX1 and ITS markers in combination allowed robust molecular identification of the isolates, and deposition of these sequences in GenBank ensures their accessibility for future comparative studies. The evolutionary relationships among species within the family Ancylostomatidae were inferred based on mitochondrial COX1 gene sequences. All eight *A. caninum* sequences from dogs in this study clustered within a subgroup of *A. caninum* sequences from other geographic regions, including isolates from dogs in the USA, Mexico, Kenya, and Australia [56], indicating a close genetic relationship and suggesting potential global circulation of this zoonotic species. *A. caninum* clustered on a basal branch together with *A. ceylanicum* [57], *A. tubaeforme* [14], and *A. duodenale* [58] from various geographic regions and different hosts, whereas *Uncinaria stenocephala* [5], despite originating from the same region as the investigated hookworms, formed a separate, more distant branch, reflecting its greater genetic divergence. The occurrence of *A. caninum*, compared to that of *U. stenocephala*, is more frequent in countries in Africa, Australia, Asia, and America, where temperatures and climate conditions significantly contribute to their continuous presence and spread [81,82,83,84]. An example of this is a case from Brazil, where *A. caninum* was identified, by applying molecular methods, in 61.4% of faecal samples from dogs [85]. Another case was reported from the USA—out of 252 samples collected from shelter dogs in Mississippi, *A. caninum* was detected in 44.4%, while *U. stenocephala* was detected in 0.8% by applying the qPCR method [86].

In the present case, no human infections associated with cutaneous larva migrans (CLM) were detected. However, several autochthonous CLM cases linked to hookworm infections have been reported in Europe. First reports of CLM came from the UK; it was a case of a 10-year-old child with 3 cm long serpiginous tracks in the metatarsal region. A similar case was also detected in a man in France—he presented with serpiginous tracks on his leg. Three additional CLM cases were reported from Italy; and in the years 2011–2019, five autochthonous CLM cases were reported from France [29,87,88]. The latest CLM report came from Poland—a case of serpiginous tracks found in three patients on their thighs and feet after they returned from Thailand [89]. Available publications did not specify the species—neither morphological nor molecular identification was presented; therefore, an analysis of a potential correlation between the CLM, *A. caninum*, *U. stenocephala*, or other species of the *Ancylostoma* genus requires further expertise. Identification of a particular hookworm species that causes CLM is demanding since it is not always possible to obtain a larva from the creeping eruption; in most cases, larvae migrate further in the epidermis at that time [90]. Only a few cases have been described with the successful collection of larvae through skin scraping or biopsy. Miller et al. (1991) stated that the larva detected in the skin lesion biopsy was identified as *A. caninum* based on its morphological characteristics [91]. In another study, three larvae were obtained through scraping the skin with follicular lesions, while the DNA was isolated from two larvae. By amplifying the ITS2 region and subsequent sequencing, a 100% identity with *A. braziliense* was confirmed. The study was the first ever molecular confirmation of the species that caused CLM [92].

The approved therapy of *A. caninum* is the one combining fenbendazole, milbemycin, moxidectin, and pyrantel active substances and potentially a combination of pyrantel, febantel, and praziquantel [21]. The patient described in this paper was administered the therapy with a combination of these three active substances. As mentioned above, diarrhoea was resolved, but the infection persisted even after the first therapy was completed; therefore, therapy with fenbendazole combined with fenbendazole, pyrantel, and praziquantel was initiated at the prescribed dose for a period of three days. Although the coprological examination of faeces was negative and the patient recovered, repeated coprological examination and molecular analysis of the third stage (L3), conducted 6 months later, again confirmed the presence of *A. caninum*. Repeated therapy with a combination of these three active substances resulted in a complete recovery of the patient, which was also confirmed by three subsequent repeated microscopic examinations (10 days, 2, and 5 months after therapy) of the faeces, all of them with a negative result. The therapy continued at home, and no adverse effects have been observed. Over the last few years, there has been an increase in the number of reported cases of patients infected with *A. caninum* in the United States, while the therapy with multiple doses or even multiple classes of anthelmintics has proved to be inefficient [21]. First reports of the potential resistance of *A. caninum* to an active substance, in particular pyrantel pamoate, appeared in 1987 in Australian greyhounds [93]. Resistance of *A. caninum to* the pyrantel active substance was reported for the first time from Australia in 2007 [94]. Moreover, several cases of resistance to fenbendazole and macrocyclic lactones were also reported [21]. In their study, Jimenez Castro et al., 2019, examined the levels of egg excretion during the therapy with fenbendazole; their interesting finding was that the number of eggs found in dogs infected with *A. caninum* first decreased by over 99%; however, after the therapy, it increased to relatively high levels [21]. The challenges associated with the therapy’s success and management of *A. caninum* are also related to the complexity of their developmental cycle [93]. Larval stages are capable of migrating to various tissues and become hypobiotic; then they are capable of encystation and reactivation, through which they move into the gastrointestinal tract again, where they develop into adults [16]. Active substances that exhibit low absorbability in the gastrointestinal tract can only affect adult individuals, but not hypobiotic larvae; this may eventually result in reinfection. If such larvae become resistant to any of the active substances, and in the case of vertical transmission to their offspring, potential resistance may become a threat to which other dogs will be exposed [21,93]. Unlike pyrantel, for example, moxidectin is highly lipophilic; therefore, it remains in tissues for longer periods of time [95].

According to a territorial study of climate change, conducted in Slovakia, global warming may be manifested in our territory in an increase in the average air temperature by 2–4 °C by 2075 [96]. In this context, it is especially significant that *A. caninum* has been detected in a 1.5-year-old female dog in Slovakia—the first case ever in Central Europe, unambiguously confirmed by applying morphological and molecular analyses. The reinfection supports the possibility that hypobiotic larvae survived either in the host or eggs and larvae (L1–L3) in the local environment, remaining capable of completing their development once conditions became favourable. Due to the facts stated above, as well as the consequences of the infections caused by hookworms for the veterinary and public health, it is important to conduct continuous monitoring of the occurrence of hookworms in dogs in Slovakia and the entire Europe. In cases of infections caused by nematodes of the family Ancylostomatidae, a follow-up coprological examination after therapy is advisable due to the possibility of reinfection. In addition, a haematological examination is recommended to detect potential eosinophilia or anaemia associated with hookworm infection. Proper hygiene measures, including the regular removal and safe disposal of dog faeces, should be emphasised to prevent the development of infective L3 larvae in the environment and to reduce the risk of cutaneous larva migrans (CLM) in humans. With ongoing global warming, caused by climate change, there is a possibility that other species of the *Ancylostoma* genus, which had until now been absent in the central and northern parts of Europe, expand into these regions. That may have a significant impact on local ecosystems, the epidemiological situation, and the management of parasitic diseases in animals.

## Data Availability

Data are contained within the article.
